# Missense *BICD2* variants in fetuses with congenital arthrogryposis and pterygia

**DOI:** 10.1038/s41439-024-00290-z

**Published:** 2024-08-26

**Authors:** Layla Masuda, Akihiro Hasegawa, Hiromi Kamura, Fuyuki Hasegawa, Michihiro Yamamura, Kosuke Taniguchi, Yuki Ito, Kenichiro Hata, Osamu Samura, Aikou Okamoto

**Affiliations:** 1 Department of Obstetrics and Gynecology, The Jikei University School of Medicine, Tokyo, Japan; 2 Department of Maternal–Fetal Biology, National Research Institute for Child Health and Development, Tokyo, Japan; 3 Center for Clinical Genetics, National Center for Child Health and Development, Tokyo, Japan; 4 Department of Human Molecular Genetics, Gunma University Graduate School of Medicine, Gunma, Japan

**Keywords:** Motor neuron disease, Genetic testing

## Abstract

Type 2 spinal muscular atrophy with lower extremity dominance (SMALED2) is caused by bicaudal D cargo adaptor 2 (*BICD2*) variants. However, the SMALED2 genotype and phenotype correlation have not been thoroughly characterized. We identified de novo heterozygous *BICD2* missense variants in two fetuses with severe, prenatally diagnosed multiple arthrogryposis congenita. This report provides further insights into the genetics of this rare disease.

Spinal muscular atrophy (SMA) with lower extremity dominance (SMALED), a rare SMA type, is characterized by musculature weakness and atrophy due to motor neuron dysfunction predominantly affecting the lower extremities and is caused by one of two autosomal dominantly inherited genes. SMALED2 (OMIM number 615290), a SMALED subtype, is caused by mutations in bicaudal D cargo adaptor 2 (*BICD2*) at 9q22.3^1^. Forty-five disease-causing *BICD2* variants have been reported in the PubMed database. SMALED2A (OMIM number 615290) is a classical form with childhood-to-adulthood onset of muscular weakness and atrophy, predominantly affecting the lower extremities. SMALED2B (OMIM number 618291) is a more severe form with prenatal onset, congenital myopathy, and arthrogryposis^1^. The correlations between the genotypes and phenotypes of SMALED2 remain unclear. We encountered two patients with *BICD2* de novo variants associated with severe multiple arthrogryposis congenita. Here, we present genetic variant information and the phenotypic prenatal course of the disease.

In Case 1, a 24-year-old nulliparous pregnant woman without significant medical or family history was referred to our hospital for a fetus with multiple fetal arthrogryposis at 17^+6^ weeks of gestation (Fig. 1a). Fetal ultrasonographic findings revealed flexed fetal wrist and elbow joints, adducted and flexed hip joints, extended knee and foot joints with talipes equinovarus, and no fetal movement (Fig. 1b and c). Despite thorough counseling, she chose to terminate the pregnancy at 19^+6^ weeks of gestation.

**Fig. 1:**
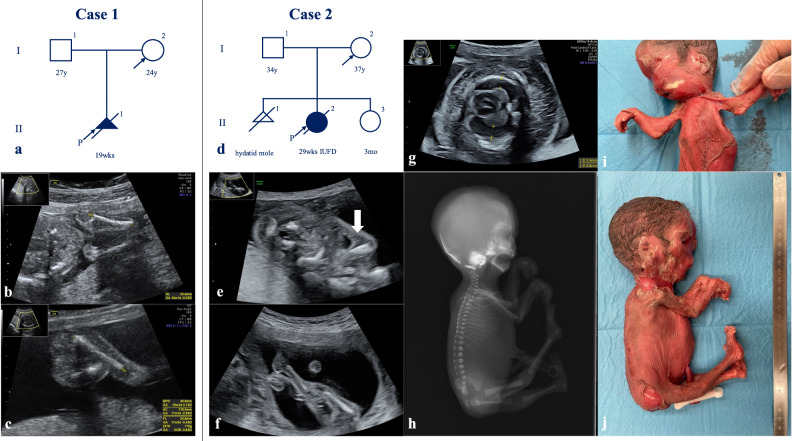
Pedigree of Cases 1 and 2 and prenatal and postnatal images of affected fetuses. **a** Pedigree of Case 1. **b**, **c** Prenatal ultrasonographic images of Case 1 at 18^+2^ weeks of gestation. **d** Pedigree of Case 2. **e**, **f** Prenatal ultrasonographic images of Case 2 at 18^+1^ weeks of gestation. The arrow indicates the pterygium at the elbow joints. **g** Prenatal ultrasonographic images of Case 2 at 23^+1^ weeks of gestation. **h** Postnatal autopsy radiograph of Case 2. **i**, **j** Postnatal gross images of Case 2.

In Case 2, a 36-year-old nulliparous pregnant woman with no significant medical or family history was referred to our hospital for a fetus with multiple fetal arthrogryposis at 18^+1^ weeks of gestation (Fig. 1d). Fetal ultrasonographic findings revealed no fetal movement, fixed and extended knees and ankles, adducted and flexed hip joints, and fixed upper extremities with flexed wrists and elbow joints. Pterygia was identified in both elbow joints (Fig. 1e and f). Single-nucleotide polymorphism microarray analysis using an amniocentesis sample revealed no chromosomal abnormalities. Fetal hydrops, subcutaneous edema, pleural effusion, and ascites developed at 20^+1^ weeks of gestation. The status of fetal hydrops gradually worsened (Fig. 1g), and intrauterine fetal demise (IUFD) was confirmed at 28^+1^ weeks of gestation. Labor was induced at 29^+5^ weeks of gestation. Autopsy radiographs revealed no abnormal findings in skeletal structures, deformities, shortening of bones, or calcification of the joints (Fig. 1h). The gross findings included multiple pterygia on the neck, bilateral axilla, cubital, and hip joints; hyperextended knee joints; and arthrogryposis (Fig. 1i and j).

We performed trio whole-exome sequencing in both cases after approval from the Institutional Review Boards of Jikei University School of Medicine and the National Center for Child Health and Development (IRB numbers: 27-060 [7945] and 926, respectively). DNA was extracted from fetal umbilical cords and parental peripheral blood. Whole-exome libraries were prepared and sequenced using a previously described method^2^. We extracted variants with minor allele frequencies <0.01 in the Integrated Japanese Genome Variation Database (ToMMo [iJGVD] 3.5KJPN; https://jmorp.megabank.tohoku.ac.jp) and the Human Genetic Variation Database (HGVD; https://www.hgvd.genome.med.kyoto-u.ac.jp) and a CADD Phred score >20. We also selected candidate variants that had a read coverage threshold >8. We identified a novel heterozygous *BICD2* missense mutant variant (NM_001003800.2:c.2200A>G, p.Lys734Glu) in the Case 1 fetus and a *BICD2* mutant variant (NM_001003800.2:c.2081G>A, p.Arg694His) in the Case 2 fetus. Neither variant was detected in the respective parents. Neither variant has been reported in any control genome database, such as the International Genome Sample Resource (https://www.internationalgenome.org), HGVD, or ToMMo (iJGVD). In silico analyses predicted a deleterious effect on protein function for p.Lys734Glu (Case 1) (SIFT, 0.002; PolyPhen2 HDIV, 0.969; PolyPhen2 HVAR, 0.793; and CADD score, 29.5) and p.Arg694His (Case 2) (SIFT, 0; PolyPhen2 HDIV, 1; PolyPhen2 HVAR, 0.999; and CADD score, 34). According to the American College of Medical Genetics and Genomics and the Association of Molecular Pathology guidelines, both variants were classified as “likely pathogenic” (PS2, PM1, and PM2 for Case 1 and PS2, PM1, PM2, and PP3 for Case 2)^3^. Therefore, the fetuses were diagnosed with SMALED2B caused by de novo *BICD2* missense mutations.

Figure 2 shows *BICD2* single-gene variants and the clinical symptoms reported as SMALED2B^4–11^. Most of the variants (94%) were associated with arthrogryposis congenita with decreased or absent fetal movement. Fetal hydrops and multiple pterygia were observed in a few cases. Ten fetuses that survived until delivery developed permanent respiratory insufficiency, with the exception of one neonate who required respiratory support for only 3 weeks. Multiple arthrogryposis congenita and decreased fetal movement were observed in Cases 1 and 2. Talipes equinovarus was detected only in Case 1. Cases 1 and 2 both followed a disease course similar to those of patients with other SMALED2B variants. Talipes equinovarus and multiple pterygia may present minor phenotypic differences among several *BICD2* variants.

**Fig. 2:**
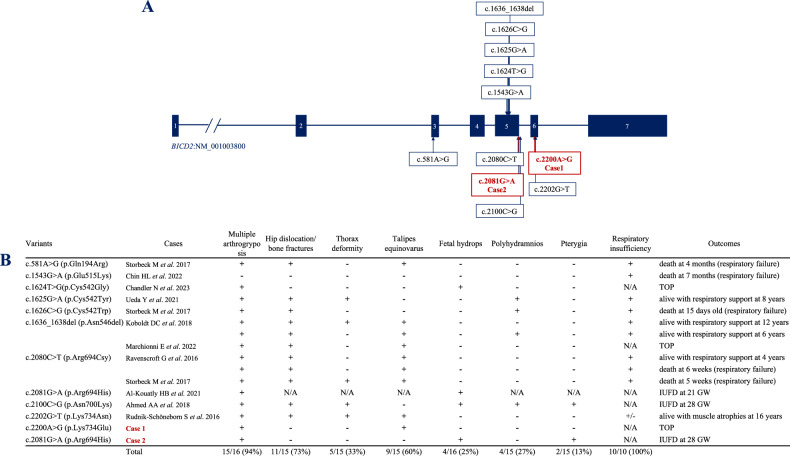
BICD2 single gene variants with clinical symptoms reported as SMALED2B. **A** *BICD2* single gene variants with clinical symptoms reported as SMALED2B, including the two present cases. The numbers in the solid boxes represent the exon number in the *BICD2* gene. **B** Table comparing the clinical symptoms of *BICD2* variants causing SMALED2B. TOP termination of pregnancy, GW gestational week, IUFD intrauterine fetal demise.

In ClinVar, an in-frame deletion variant at the same location as the variant in Case 1, NM_001003800.2 (*BICD2*):c.2200_2202del (p.Lys734del) (Variation ID: 982816), is registered as likely pathogenic and causes childhood-onset proximal spinal muscular atrophy with contractures. A previous study reported another disease-causing missense variant, c.2202G>T (p.Lys734Asn), at a location close to that of the variant in Case 1 (Fig. 2B)^12^, resulting in a prenatal onset phenotype with multiple congenital articular contractures with leg and thorax deformities and multiple muscular atrophy. However, this patient survived for >16 years, confirming a relatively milder form of SMALED2B caused by this variant compared with other variants. Since the pregnancy was terminated in Case 1, a thorough comparison of the clinical course of these three variants, Lys734Glu, Lys734Asn, and Lys734del, is difficult. The disease onset at the 17th week of gestation with severe multiple arthrogryposis in Case 1 might have been associated with a relatively more severe course among the three variants. The Lys734 codon is located in the coiled-coil 3 region, which interacts with RANBP2 and RAB6, indicating the important function of *BICD2*. Further clinical cases need to be studied to define the phenotype of variants at this amino acid.

A single previous study reported the variant c.2081G>A (p.Arg694His), the same variant detected in Case 2 (Fig. 2B); fetal hydrops with akinesia was detected at 19 weeks of gestation, and labor was induced owing to IUFD at 21 weeks of gestation. However, the clinical course was not detailed in the report^13^. This article details the clinical symptoms and outcomes of this variant, which follows a course similar to that reported previously. A total of four cases were previously reported with variants located at the same amino acid, p.Arg694^4,10,13^, as in Case 2. All patients with the p.Arg694Cys variant survived until live birth but developed respiratory insufficiency. However, fetuses with p.Arg694His, including the fetus in Case 2, developed fetal hydrops and did not survive until birth. The Arg694 codon is also located in the coiled-coil 3 region, which interacts with RANBP2 and RAB6, indicating the important function of *BICD2*.

In conclusion, we presented *BICD2* missense mutation variants from two independent fetuses with severe multiple arthrogryposes. We highlight the importance of reporting new cases to provide further insights into the genetic basis of this rare disease.

## HGV database

The relevant data from this Data Report are hosted at the Human Genome Variation Database at 10.6084/m9.figshare.hgv.3427, 10.6084/m9.figshare.hgv.3430, and 10.6084/m9.figshare.hgv.3246.

## Supplementary information

Supplementary Table

Supplementary Table
