# Supplementary material for: Missense BICD2 variants in fetuses with congenital arthrogryposis and pterygia
Source: Hum Genome Var. 2024 Aug 26;11:32. doi: 10.1038/s41439-024-00290-z (PMC11345410; doi:10.1038/s41439-024-00290-z)
Supplement: Supplementary file 1 — Supplementary Table [file 41439_2024_290_MOESM1_ESM.pdf]

Case 1

Gene	Codon	Protein	SIFT	PolyPhen2 HVAR	CADD score	jMorp	Father	Mother	Proband	Zygosity
BICD2	c.2200A>G	p.Lys734Glu	D	P	29.5	NA	0|0	0|0	0|1	de novo hetero
CNTRL	c.6221C>A	p.Ala2074Asp	T	B	21.4	0.0013	0|1	0|1	1|1	homo
NEB	c.14411G>A	p.Arg4804His	T	B	22.5	0.0048	0|0	0|1	0|1	compound hetero
NEB	c.10303A>G	p.Met3435Val	T	B	23	NA	0|1	0|0	0|1	compound hetero
ZBBX	c.1015C>T	p.Pro339Ser	D	B	22.8	0.0628	0|0	0|1	0|1	compound hetero
ZBBX	c.439C>T	p.Leu147Phe	T	B	22.5	0.0004	0|1	0|0	0|1	compound hetero
PRRC2A	c.6016C>T	p.Pro2006Ser	D	P	24.2	0.0035	0|0	0|1	0|1	compound hetero
PRRC2A	c.6278C>G	p.Thr2093Arg	D	B	22.8	NA	0|1	0|0	0|1	compound hetero
GPR179	c.2650C>T	p.Arg884Trp	-	B	20.6	NA	0|1	0|0	0|1	compound hetero
GPR179	c.1021G>A	p.Gly341Arg	-	B	20.7	0.0011	0|0	0|1	0|1	compound hetero
LAMA5	c.5624C>T	p.Pro1875Leu	T	D	24.1	NA	0|1	0|0	0|1	compound hetero
LAMA5	c.3055G>A	p.Glu1019Lys	D	P	34	NA	0|1	0|0	0|1	compound hetero
LAMA5	c.2665G>A	p.Val889Met	T	P	23.4	0.0292	0|0	0|1	0|1	compound hetero

Case 2

Gene	Codon	Protein	SIFT	PolyPhen2 HVAR	CADD score	jMorp	Father	Mother	Proband	Zygosity
FAM13A	c.1288C>G	p.Arg430Gly	D	D	29.7	NA	0|0	0|0	0|1	de novo hetero
BICD2	c.2081G>A	p.Arg694His	D	D	34	NA	0|0	0|0	0|1	de novo hetero
GIGYF2	c.3611_3614insCGCAACA	-	-	-	-	NA	1|2	1|2	1|1	homo
MEF2A	c.1027_1032del	p.343_344del	-	-	-	NA	0|1	1|2	1|1	homo
CES1	c.1087-2_1087-1insT	-	-	-	-	NA	0|1	0|1	1|1	homo
PLEKHH2	c.1423G>A	p.Ala475Thr	T	D	23.7	0.0001	0|0	0|1	0|1	compound hetero
PLEKHH2	c.3958C>A	p.Leu1320Ile	T	P	24.6	0.0016	0|1	0|0	0|1	compound hetero
TTN	c.78325C>T	p.Arg26109Cys	D	P	24.9	0.0023	0|0	0|1	0|1	compound hetero
TTN	c.71270A>G	p.Asp23757Gly	D	D	20.4	0.0004	0|1	0|0	0|1	compound hetero
TTN	c.42359G>C	p.Arg14120Pro	D	D	23.1	NA	0|1	0|0	0|1	compound hetero
TTN	c.37897C>T	p.Arg12633Cys	T	D	23.7	0.0092	0|1	0|0	0|1	compound hetero
TTN	c.9038A>T	p.Glu3013Val	T	P	23.4	0.0004	0|1	0|0	0|1	compound hetero
