# Supplementary material for: Missense BICD2 variants in fetuses with congenital arthrogryposis and pterygia
Source: Hum Genome Var. 2024 Aug 26;11:32. doi: 10.1038/s41439-024-00290-z (PMC11345410; doi:10.1038/s41439-024-00290-z)
Supplement: Supplementary file 2 — Supplementary Table [file 41439_2024_290_MOESM2_ESM.docx]

**Supplementary Table:** All candidate variants after whole-exome sequencing analysis of Cases 1 and 2. Variants with minor allele frequencies <0.01 in the Integrated Japanese Genome Variation Database (ToMMo [iJGVD] 3.5KJPN; https://jmorp.megabank.tohoku.ac.jp) and the Human Genetic Variation Database (HGVD; https://www.hgvd.genome.med.kyoto-u.ac.jp) and a CADD Phred score >20 were extracted. We also selected candidate variants that had a read coverage threshold >8.
